# How Does the Campus Environment Influence Everyday Physical Activity? A Photovoice Study Among Students of Two German Universities

**DOI:** 10.3389/fpubh.2020.561175

**Published:** 2020-10-05

**Authors:** Julia von Sommoggy, Jana Rueter, Janina Curbach, Jessica Helten, Susanne Tittlbach, Julika Loss

**Affiliations:** ^1^Medical Sociology, Department for Epidemiology and Preventive Medicine, University of Regensburg, Regensburg, Germany; ^2^Social and Health Sciences in Sport, Department of Sport Science, University of Bayreuth, Bayreuth, Germany

**Keywords:** photovoice, physical activity, University campus, sedentary behavior, setting approach, Health Promoting Universities, students

## Abstract

**Background:** Sedentary time is high among university students. Prolonged sitting time and reduced physical activity is linked to a number of health risks, therefore interventions to increase options for physical activity on campuses are of high public health relevance. Evidence about the influence of the campus environment on movement and sedentary behavior of students is scarce. This study explores how the structural and social environment of two University campuses are related to students' everyday physical activity.

**Methods:** We used the photovoice method to get a thorough insight into students' daily life on campus. We recruited a total of 46 University students in two German cities (University 1: *n* = 22, University 2: *n* = 24). They were asked to take ≥15 photos of places and situations on their respective campus that facilitate or hinder them to be physically active. The pictures were discussed with the participants in 10 focus groups. Focus group discussions were audio-recorded, transcribed and analyzed using content analysis.

**Results:** Both universities do not exploit their potential of fostering daily physical activity on campus, according to the photos and discussions of the participating students. The vast green spaces offer no cues for movement: easily accessible equipment for sports (fixed or mobile) is lacking, walkways are partially hidden, and the facilities discourage from cycling to and on campus. Social norms induce participants to keep sitting during lectures and learning time. It was also pointed out that indoor hallways and foyers could be put to better use with regard to physical activity. The Photovoice project raised the participants' awareness of how the context influences their movement behavior, and helped them come up with solutions to make physical activity easier for students of their respective universities.

**Conclusion:** The studied campuses discouraged students from being physically active by missing out on opportunities—indoors and outdoors—for fostering movement, such as designating the greens for games or walks, or providing sufficient lockers for biking gear. The results can serve as a basis to plan custom-made public health interventions.

## Introduction

Sedentary behavior has been identified as a major health risk. There is a growing number of studies indicating a relationship between prolonged sitting time and health risks, such as an increased risk for all-cause mortality, in addition to several chronic conditions, e.g., diabetes, different cancers, or cardiovascular disease (1). Germany is among those countries with a fairly high sitting time (2). In adults, higher socioeconomic status is related to prolonged sitting in the workplace (3, 4). Within universities, sedentary behavior has been a social norm which is ingrained in most typical procedures, structures, and behavioral patterns of these institutions: Students are sitting during lectures, in seminars and in the library, additionally during lunchtime, in the cafeteria and often during breaks between sessions. Sedentary time encompasses on average 34 h per week for University students while staying on the campus, and this does not yet include (passive) transportation, study time at home or leisure time (5). Only 50% of young adults in Germany are physically active for 150 min or more per week, as is recommended by the World Health Organization (6).

It is therefore important to increase options for movement in the daily campus life, in order for students to be able to reduce their sedentary time. Studies from other settings, e.g., schools, show that environmental contexts shape children's and adolescents' everyday life and are likely to influence their (health) behavioral patterns. Quantitative research has shown, for example, that physical activity of students is positively correlated with a larger total school campus size, playground areas and facilities for physical activity (7–10). Few qualitative studies on that subject shed light on the potential ambivalence of these correlations, as some students reported that they were “too old” for playgrounds and considered “safe play spaces boring” (10).

Studies on structural barriers and facilitators for physical activity experienced by University students are scarce. Arzu et al. showed that the perceived lack of time is a major barrier for physical activity among University students (11). Apart from that, other barriers or facilitators for movement in the University environment have hardly been investigated yet. Understanding the contextual influences that shape sedentary or movement behavior of University students is critical for planning needs-based interventions. Evaluations of interventions that aimed at increasing physical activity among University dwellers show that these approaches tend to be effective, but were mainly conducted in US American colleges, which show significant differences compared to German universities (12). It becomes clear that we need to better understand how the environments within which University students spend a lot of their time might act to enhance or constrain sedentary behavior and physical activity (13).

Therefore, this study intended to explore and understand how factors of the structural and social environment in University campuses influence physical activity and sedentary behavior of students. We chose the Photovoice technique, a participatory action research first described by Wang and Burris (14, 15). In Photovoice, the participants are provided with cameras (or use the photograph function of their mobile phones) to identify and document resources, barriers and facilitators with regard to a certain topic or behavior (e.g., food security, physical activity, living with HIV) in their surroundings. Participants are then brought together as a group in order to discuss a selection of the photos taken. These discussions contextualize the photographic data, and have the participants identify common themes and concepts. This process is not only useful to researchers or health promoters, but may also benefit the participants. They may develop critical awareness of their environment and the role that this environment plays in influencing behavior patterns (16). The Photovoice technique is based on Paulo Freire's theories on participatory education, and Feminist theory focusing on giving voice to the disadvantaged (14). It helps people use visual evidence to recognize and voice their problems and potential solutions to researchers, which in turn communicate these concerns and suggestions to policy makers (16). The ultimate aim of the Photovoice technique is to bring about social change in a setting. Since its inception, the Photovoice methodology has been applied to a variety of populations, places, health issues, and disciplines. Some studies have performed Photovoice with adolescents, in order to capture their perspectives on health and well-being (17), healthy eating and active lifestyle barriers (18), or sexual health information, alcohol and drugs (19).

We performed a Photovoice project intending

- to actively engage University students in documenting and discussing their campus surroundings and daily lives with regard to physical activity and sedentary behavior
- to explore factors in the campus context that hinder or facilitate physical activity and active living

## Materials and Methods

### Context of the Photovoice Study

We performed a Photovoice study with students from two German campus universities [total number of students enrolled University 1 (U1): 13,500 and University 2 (U2): 21,000] from May to July 2018. In these two universities, researchers had received funding to implement campus-based measures that promote and facilitate physical activity for students (project “Smart Moving”), and intended to found stakeholder groups with students, lecturers and University administration staff to plan these measures. The Photovoice study was meant to obtain an insight into the daily life of the students, and into the role that the campus context plays in their physical activity. Thereby, we intended to obtain ideas for adequate interventions (=needs assessment) which could inform the stakeholder groups. It was also meant to raise the participants' critical awareness of environmental influences on their movement behavior, and thereby also possibly motivate some participants to take part in further activities (i.e., recruiting them for the stakeholder group).

### Proceedings of Photovoice Project

#### Research Participants

In qualitative research, a sample's statistical representativeness is not a prime requirement, especially when the research aims at understanding social processes (20). A strategy to ensure rigor in qualitative data collection is using systematic, or purposive sampling, e.g., theoretical sampling, by identifying specific groups of people who possess certain characteristics that are relevant to the social phenomenon being studied (20). In our study, we strove at including (1) a comparable number of male and females in the sample, as views on physical activity may be gender-related, (2) a broad range of different study programs that participants were enrolled in, in order to obtain a balanced composition with regard to expertise, areas usually frequented during study time, and time schedules. Thus, we intended to minimize bias in the composition of the sample.

We recruited students at both universities with the aim of recruiting a minimum of 20 students per University. We used bulletins, flyers, University mailing lists, personal contacts and a snowballing technique.

Participants had to fulfill the following criteria: They needed to be (a) enrolled students of the respective universities, (b) able to take digital pictures, and (c) willing to participate in a 1–1.5 h focus group discussion.

The recruitment was terminated when theoretical saturation was reached, i.e., no more new themes came up in the focus group discussions (see below).

#### Sample

We recruited 22 students (14 female, eight male) in University 1, and 24 students (14 female, 10 male) in University 2. The average age was 23.6 ± 2.2 years (U1), and 23.8 ± 2.8 years (U2), respectively. Four students did not live within the town of the University, but on the outskirts, one student lived further away (1 h drive to and from campus). Most of the interviewees were of German nationality, one was from Tanzania. They were enrolled in 14 different study programs, ranging from tourism and history to medicine and engineering. Most frequent study subjects were psychology (*n* = 8), sport science (*n* = 6), and law, teaching and economics (each *n* = 5). (see Table 1).

**Table 1:** Sociodemographic data of the participants.

	**University 1 (*n*)**	**University 2 (*n*)**	**Total (*n*)**	**Total (*%*)**
**Total**	22	24	46	
**Study course**				
Economics	8	1	9	19.6%
Natural Sciences	9	8	17	37.0%
Law	3	2	5	10.9%
Social Sciences	2	13	15	32.6%
**Gender**				
Male	8	10	18	39.1%
Female	14	14	28	60.9%
**Age**				
20–24	9	17	26	56.5%
25+	12	7	19	41.3%
n.a.	1		1	2.2%
**Nationality**				
German	21	24	45	97.8%
Tanzania	1		1	2.2%
**Place of residence**				
Town of University	21	21	42	91.3%
Other	1	3	4	8.7%

#### Procedure

Students contacting the project team because they had learnt of the study (recruitment strategies: see above) were provided with further information. The participants received a list of specific questions (“Where and when am I physically active during my daily life on campus? What prevents me from being physically active during my daily life on campus?”), which they were asked to consider when taking their pictures. The participants were asked to take at least 15 pictures with their smartphones of places and/or situations where physical activity during their daily life was considered to be easily possible, or not/hardly possible, or made easy or difficult. They were specifically asked to include not only sports, but any physical activity (e.g., taking the stairs instead of the elevator, etc.). To ensure guidance, students were invited to contact the project team any time in case of doubts or questions. As smart phones equipped with cameras are an integral part of the University students' life, training with regard to taking pictures was deemed unnecessary.

Participants were asked to send the photos to the researchers via e-mail. The received photo files were then printed on a larger scale (13 × 18 cm) and laid out during the focus group discussion. The photos were equipped with post-its containing letters and numbers. The participants were asked to mention those letters and numbers during the focus group when talking about a specific picture in order to make the picture distinguishable for the researchers during the analysis.

We performed ten focus groups (five in each University) with 3–5 participants per group. The focus group discussions were guided by prompts (see Table 2). Based on the SHOWED proceeding recommended for the Photovoice approach, the participants were first asked to explain what can be seen on the picture, and what was happening in the photographed scene, in order to understand the specific focus of the participants (21). The participants then described how the picture connected to physical activity, and how the participants appraise this situation. For example, stairs can encourage physical activity (as opposed to elevators), but may also render physical activity difficult (when being an obstacle for using the bike). Finally, the students were asked to come up with ideas how the situation could be changed or used to make physical activity on campus easier (21, 22).

**Table 2:** Questions guiding the focus group discussions of the photographs.

Questions relating to the pictures
✓ What does the picture show? ✓ What did you want to show us with this picture? ✓ How does the picture connect with your personal everyday physical activity on campus?

After conducting three focus groups in both universities, no more novel picture motives came up. As the discussion in the focus groups evolved around the images provided by the students, the same topics were covered in the discussion. Two more focus groups were conducted in each University, to ensure theoretical saturation was reached. The recruitment was stopped subsequently (23).

#### Informed Consent and Confidentiality, Ethics Approval

The study was approved by the Ethics Committee of the University of Regensburg (18-896-101). Written consent was obtained from all study participants in accordance with the ethics approval. The participants were informed that they could opt out at any time of the process. They received an expense allowance of 45 Euro for their time spent.

#### Analysis

All focus groups were audio recorded and subsequently transcribed verbatim. All transcripts were de-identified before analysis. The focus group (FG) transcripts were numbered chronologically (FG 01 - FG 10) and classified according to respective University (U1, University 1; U2, University 2) before de-identification. Two researchers coded the transcripts independently, using content analysis (24, 25). The researchers extracted themes and topics with regard to environmental barriers and facilitators of physical activity, following an inductive approach. The transcripts were re-read repeatedly, and the themes and topics were compared between the different focus groups to identify cross-cutting themes that came up in several group discussions. The pictures were not explicitly analyzed, but taken as a reference for the text (26). They received a de-identified number according to the student who took the photo (S1-S22 in U1, S23-S46 in U2). To increase the scientific rigor of the analysis, the independent assessments of transcripts by the two researchers were compared; differences were discussed until consensus was reached. In addition, “negative” or “deviant” views were examined with specific thoroughness (20).

## Results

During the analysis, several pathways in which the University context shapes physical activity and/or sedentary behavior emerged, and different barriers and facilitators to an active lifestyle could be identified (see Table 3). The participants also came up with solutions to overcome certain barriers.

**Table 3:** Summary of results.

**Feature**	**Effect on physical activity**
**Campus outdoor**	
Green fields/lawns	+ Space for physical activity is available – Cues for physical activities are lacking − Terms of use are unclear
Design of outdoor areas/campus centers	+ Attractive walkways encourage students to take a walk – Amphitheater-like structures with steps are a barrier to movement
Sports center	+ A wide range of appealing courses are offered – A location at the margin of the campus prevents usage of sports equipment in breaks – The provided offers and options are not well communicated to students other than sports students
Signposting	– Attractive walkways or venues suitable for short walks (e.g., botanic gardens) are not signposted and difficult to find – Bikeramps to avoid the stairs on the campus are not signposted for cyclers
Bike racks	+ Roofed and sufficiently available racks encourage students to cycle to and from campus + Small stations for bicycle repair (air pump etc.)
**Campus indoor**	
Lockers	+ Can be used to store cycling gear (helmets etc) – Shortage of lockers renders the storing of biking gear difficult, which can be a reason for students not to take the bike to campus
Staircases	+ Vast, easily accessible stairs in the middle of building may nudge students to take stairs rather than elevators – Hidden staircases foster taking the elevator
Halls and foyers	– Lack encouragement/cues for movement
Library working places	+ Stand-up tables in libraries motivate students to stand – Stand-up tables are low in numbers, which makes usage difficult – Personal books and laptops cannot be stored safely and therefore not left behind, which prevents active breaks
Lecture hall design	– Long narrow rows of chairs prevent students from getting out in between lectures

### Active Transport to the Campuses Could Be Enhanced by Improving the Campus Environment for Cyclers and Their Gear

At both universities, the participants explained that the campus could easily be reached by bicycle from centers and living quarters of the respective towns. Cycling paths were provided throughout the towns, encouraging students to take the bike.

There is an old railway trail, which was tarred some time ago. It's perfect for riding the bike, because it is set aside from the road. For me, that is a reason to take the bike to campus. You are out in the nature, and you can clear your mind. (U1 FG2)

Participants reported difficulties in parking their bikes. They especially pointed out the lack of sheltered bike racks preventing bikes from getting wet in rain in University 2.

I have a fairly good bike and I don't want it to stand in the rain all day. This really prevents me from going to the University, for example at the weekend, to study in the library. (U1 FG5)

**Figure 1:**
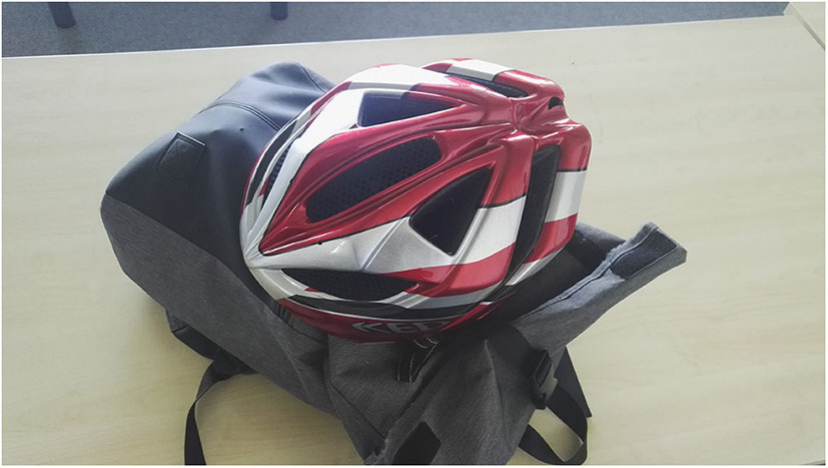
Participants reported that they needed to carry with them a lot of gear, which is difficult to store on campus (Photo: U1 Student S3).

University 1 offers a bike station in which students can pump up tire and do little repair works; this is very much appreciated by the participants and considered a factor encouraging them to bike to campus. Bad weather (e.g., rain) itself does not so much deter students from cycling to the campus, but the lack of adequate storage room for wet clothing, rain jackets etc. does, according to the focus group discussions (A: Figure 1).

**Figure 2:**
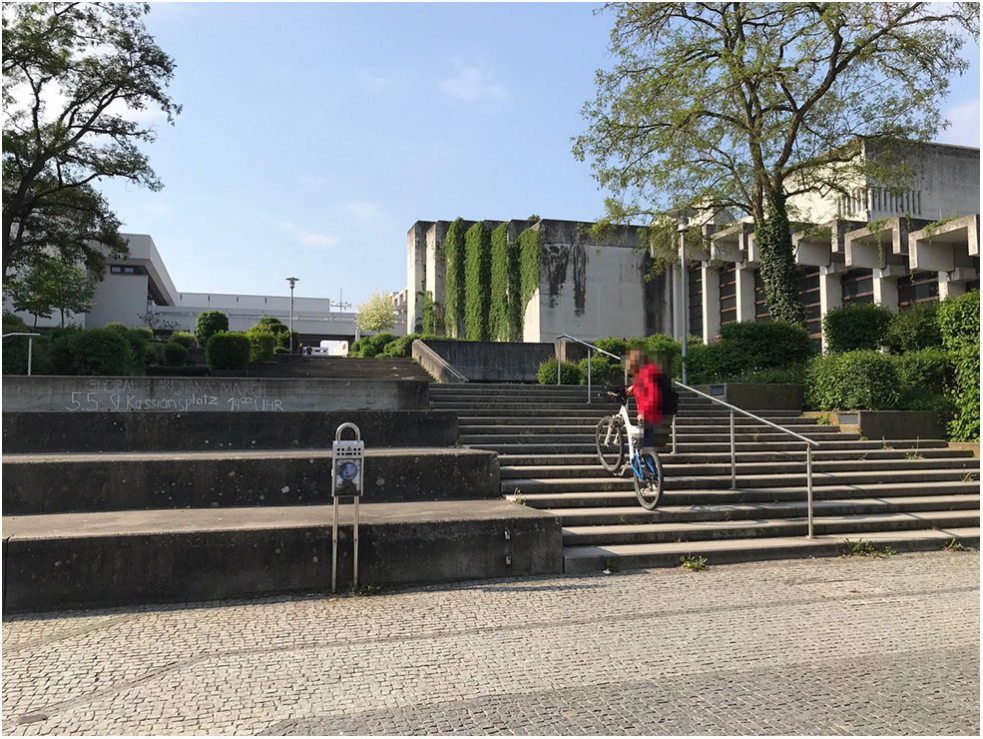
It is not clear how to circumnavigate these stairs. As there are no bike ascents, the bike has to be carried all the way up, which makes it difficult and uncomfortable to use the bike on campus (Photo: U2 S27).

In University 2, there are many stairs all over the outdoor area, and they are considered a major obstacle to riding the bike on campus (B: Figure 2). This might even prevent some students from taking the bike to campus in the first place. The participants suggested that bypasses for bikes or ramps should be indicated more clearly in order to help cyclers navigate the campus more easily.

There are ‘hidden paths’ to circumnavigate the stairs. You get to know them when you take the bike and enjoy exploring your surroundings. But you have to find out for yourself, there are no signs indicating how stairs can be circumnavigated. (U2 FG7)

### The Vast Outdoor Areas Motivate to Sit and Relax, Rather Than to Move and Be Active; Cues for Physical Activity Are Lacking

On both campuses, there are wide lawns between the buildings. Footpaths linking buildings are valued for their location within the green spaces, and are sometimes even used for detours.

I really like this path. I take it all the time with the bike or by foot. It definitely encourages physical activity for me, because it has nice surroundings. (U2 FG10)

However, in University 2, students complain that despite the vast grounds of the campus, attractive walking trails are lacking. They felt that the campus area could be put to better use if special pathways encouraged students to stroll around at different sites.

I would like to have a walkway where I can walk and contemplate, or can stroll around with other people. Somehow, this University campus does not seem inviting for taking a walk. There is no footpath with special spots along the way, e.g. inspiration for meditation or something similar. … I often feel somewhat lost on campus, even though I have been studying here for seven years. I would love to go for a walk to clear my mind sometimes, but I don't feel comfortable doing that, because I'm always afraid of getting lost, or not being back in time. (U2 FG9)

**Figure 3:**
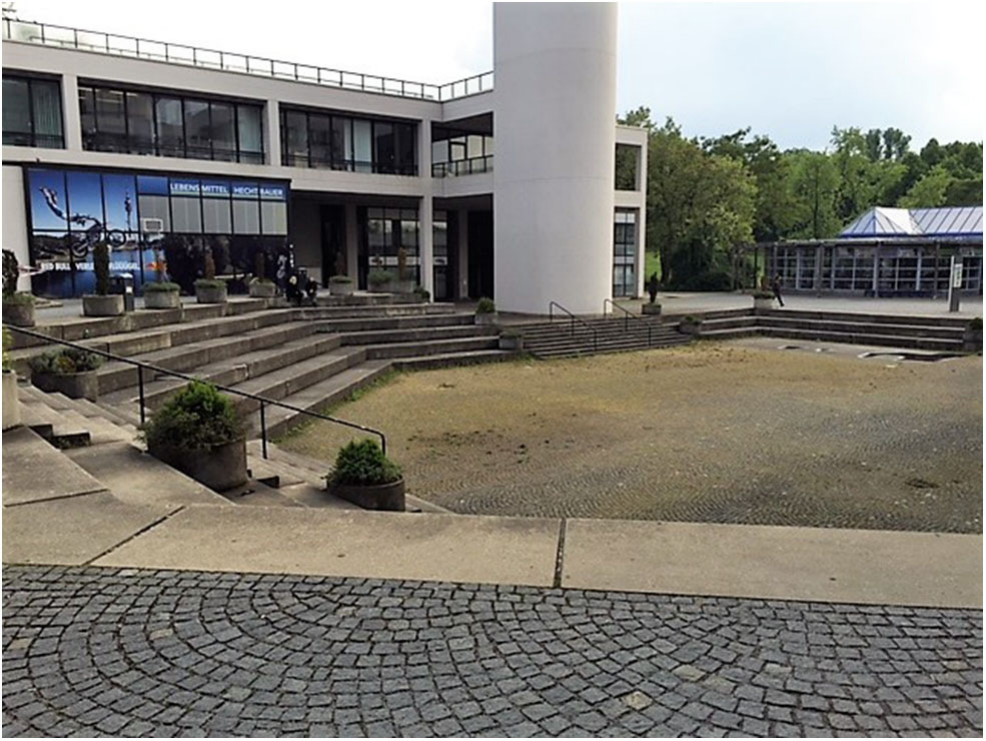
The Amphitheater is a place where students sit, rather than walk or stand. The stairs invite to sit down while eating, drinking coffee or talking (Photo: U2 S37).

University 2 also disposes of a central plastered area built in an Amphitheater style, which proves to be a barrier to physical activity: the Amphitheater stairs are used to sit rather than to walk, and people avoid moving through or within the half-oval as they feel observed by the people sitting above them (C: Figure 3).

**Figure 4:**
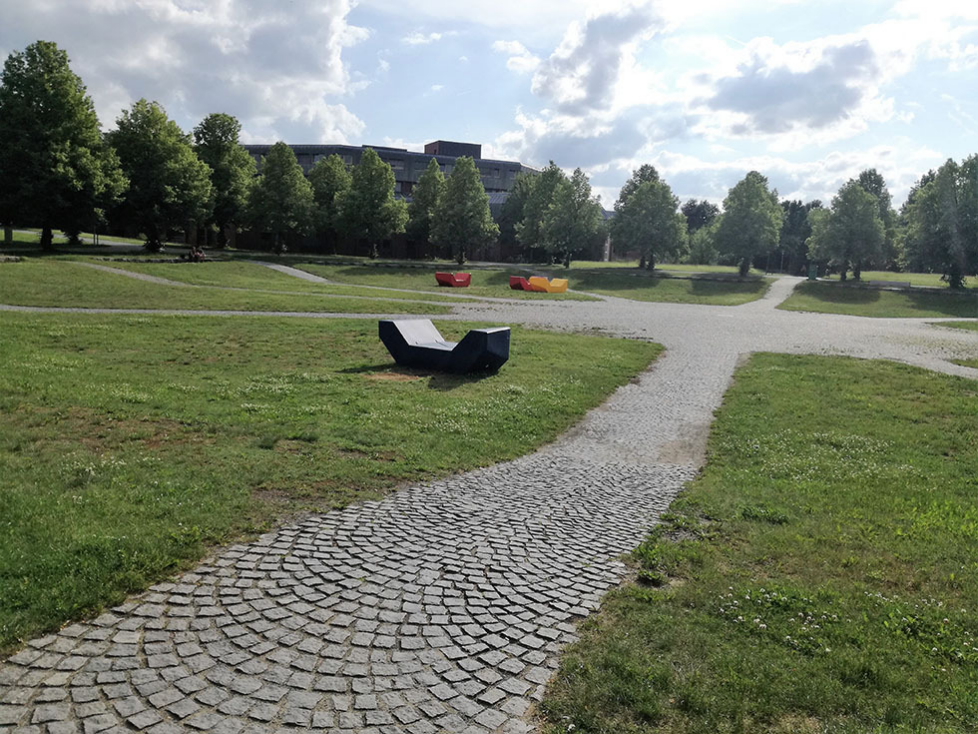
The round structure of the campus, with the faculties located on the side of the circle, are inviting to go for a walk. Shaded by trees, the students can stroll around during their break (Photo: U1 S6).

In University 1, on the other hand, there is a great circular path in the center of the campus (“rondel”) that invites students to take walks around while talking to friends, drinking coffee, or even learning (D: Figure 4).

I know some people who even take their study scripts with them and stroll around the “rondel”, for example when they need to memorize something. (U1 FG1)

The “rondel” offers a great opportunity to walk “laps”. I do that all the time and because the length is manageable and visible, you might bring yourself to walk one more than actually intended. (U1 FG 4)

**Figure 5:**
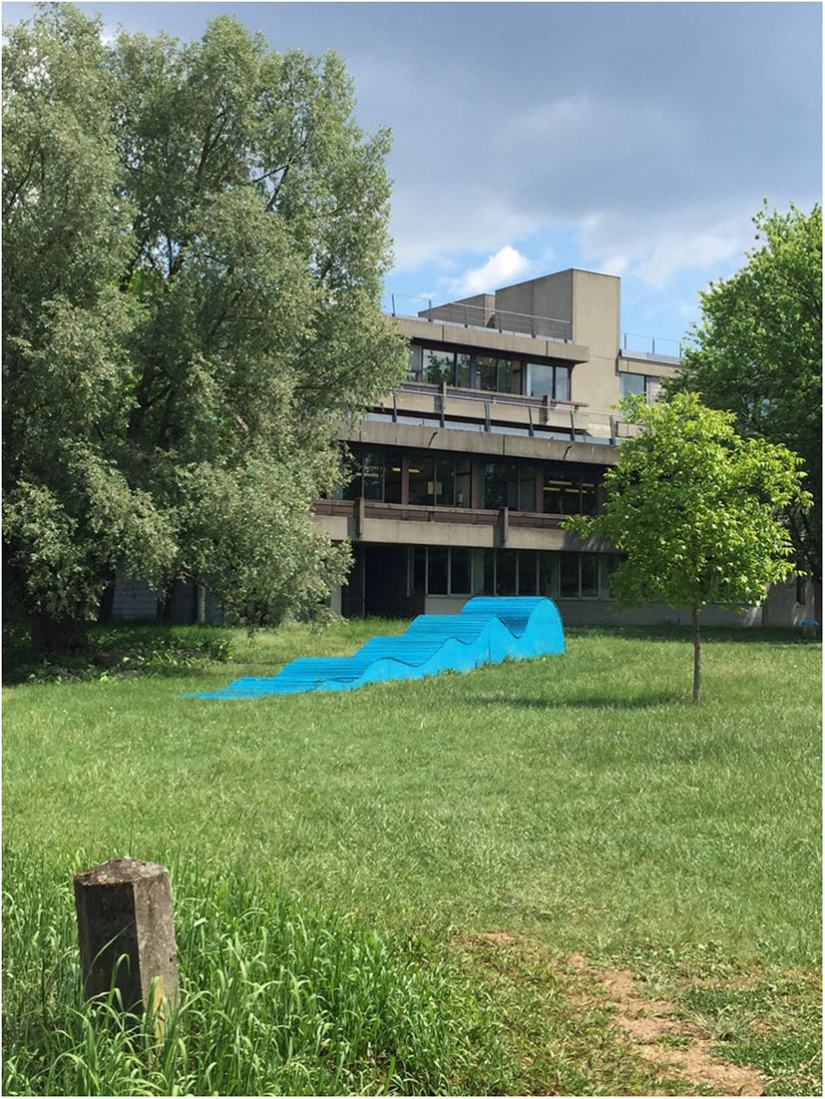
Students tend to sit or lie down on the lawn during their breaks, as there is no facility encouraging them to move (Photo: U2 S28).

Other than that, the green spaces and campus squares invite students to sit down and relax, rather than move, according to the participants (E: Figure 5).

Further barriers are uncertainties about the campuses' usage policies of the green spaces, e.g., whether (ball) games are banned.

Here's the problem: it is not clearly communicated what you are allowed to do on those vast green spaces. What is forbidden and what is permitted? Can we play soccer? (U2 FG8)

The participants regret that there is no sports equipment, e.g., balls, rackets etc. available for use or hiring. Bringing this equipment to campus is considered cumbersome due to the lack of storage options. There are opportunities for exercise available, like a slackline park or a bouldering tower, but they are situated close to the sports center at both universities. Especially at University 2, the sports center is rather separated from the rest of the campus, thus the offers are partially unknown or considered too far away to use during breaks. The participants felt that fixed outdoor movement options on the main campus (rather than in the area of the sports center), e.g., slacklines, table tennis, or table soccer, would encourage many students to be more physically active during breaks. They pointed out that easy access and playful character of these options would be beneficial.

Close to the philosophic faculty building, there are trees where you could easily install some slacklines. I really like using slacklines, I really think I would do it more often if it was closer by. I would say: “Guys, let's go and do some slacklining…” (U2 FG7)

[Possibilities for being physically active] – it is necessary for those to be known by all students. Maybe slacklines, ping pong tables. It is necessary to distribute the sports opportunities over the campus, rather than centralize everything around the sports center. (U1 FG3)

It would be great if you were able to claim: “I'm not really being physically active in the sense of doing sports, but doing something fun, which in turn entails being physically active.” (U1 FG3)

### Sports and Activity Areas Are Part of the Campuses, but Out of the Scope of Many Students

The sports centers within the universities are highly appreciated, but several aspects prevent students from making the most of the available offers, according to the participants. One reported barrier is the physical distance, especially in University 2, where the sports center is separated from the rest of the campus by a street which can be crossed via a long bridge.

The bridge separates the University campus from the sports center. Over there, there is so much movement [among the students]: people are playing Beach Volleyball, or they are slacklining. And on the other side of the bridge [on the main campus], people are just lying in the sun. (U2 FG6)

I love slacklining, but when I have a 30-minute break, it is not enough time to walk over to the sports center where the slackline park is located. It takes me 10 minutes just to get there. (U2 FG7)

The (spatial) distance is also linked to a lack of transparency about options and offers for movement that can be found in and around the sport centers, as was reported about both universities.

For me, all these sportive offers are a mystery. We don't get information about that. We simply don't know what we are allowed to use and how. I heard it was possible to rent sports equipment? (U1 FG2)

I study sports, and only I knew about this (sports) event (a fun competition in dodge ball). This event is free and you can try out everything. I thought it is so sad, on campus there are so many posters etc. about parties etc. but nothing about this sport event. You can see that physical activity is not regarded important. (U2 FG6)

### The Design of Stairs and Staircases Influences Their Use

**Figure 6:**
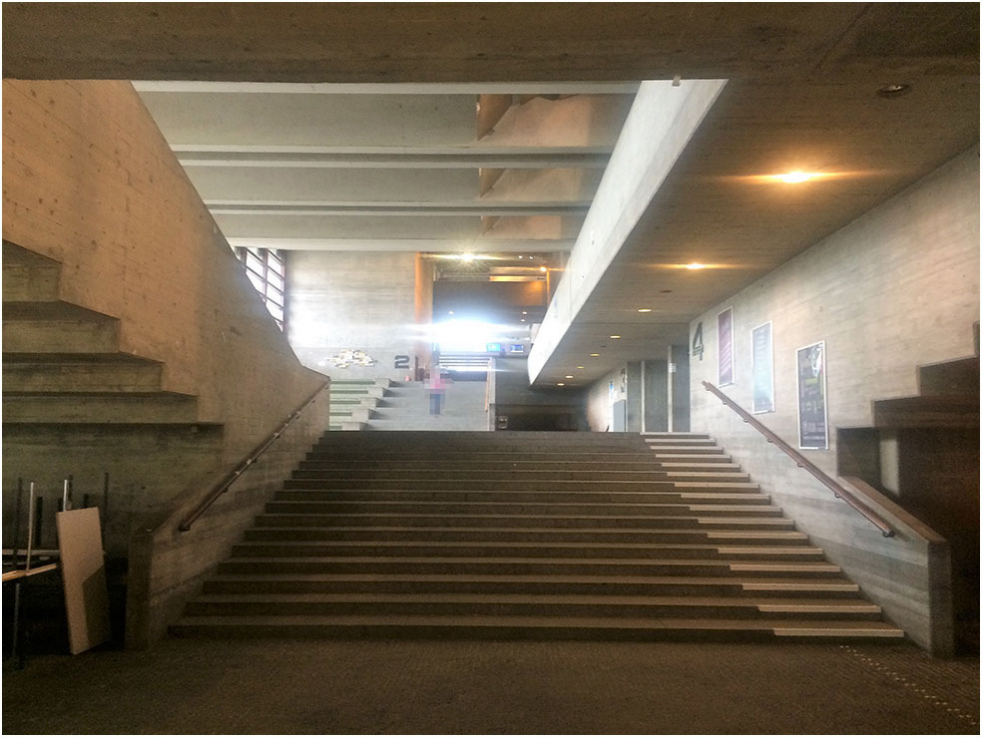
Wide staircases are inviting to use them. Students use them automatically without thinking about it (Photo: U2 S31).

When discussing pictures of the interior of the University buildings, the participants acknowledged that using the stairs is a good opportunity for physical activity. It transpired that students use stairs (instead of elevators) with different frequency in different buildings, depending on the architecture. In buildings with wide, open staircases, participants report to feel encouraged to use the stairs (F: Figure 6), or use them “automatically” without noticing. In other buildings, the staircases are hidden or less accessible, which leads to a more frequent elevator use.

In the XY building [a building with rather narrow staircases behind heavy doors], there are two elevators and I'm really the only one taking the stairs there. Sometimes people are waiting upstairs until people have ridden downstairs with the elevator and the elevator is coming up again, instead of taking the stairs! (U2 FG8)

**Figure 7:**
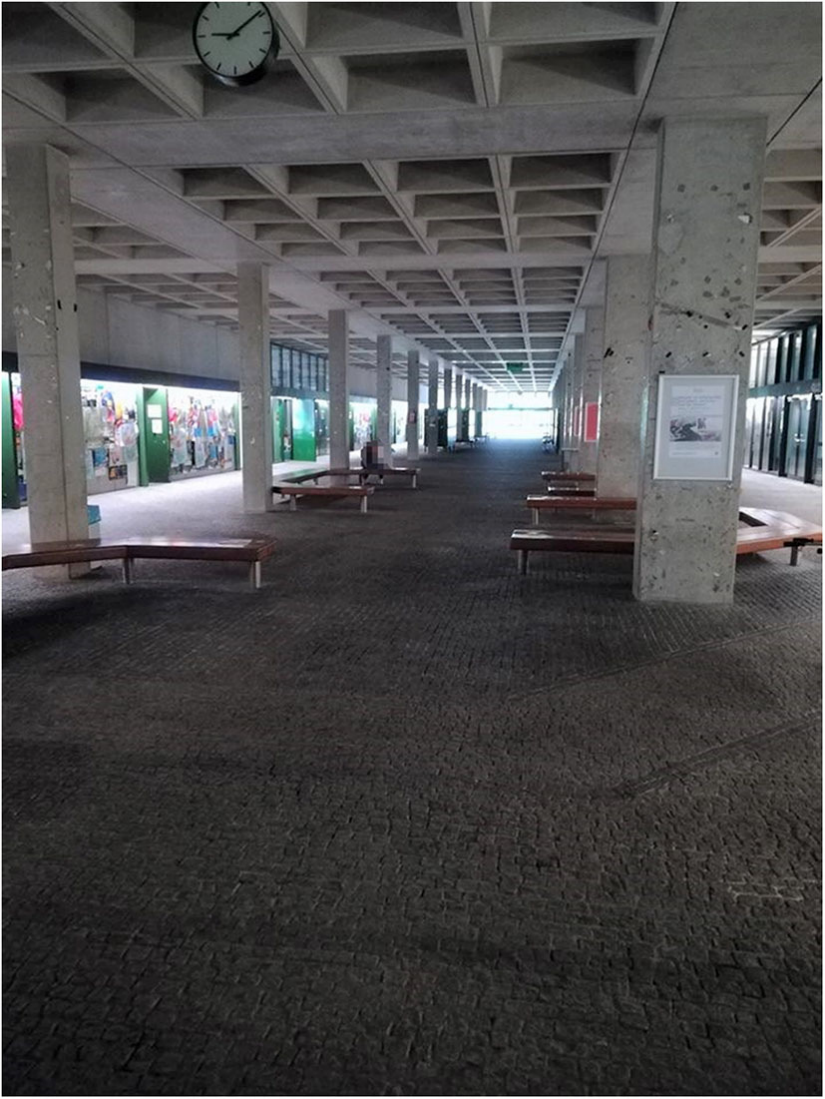
Large hallways are available all over the campus, but there is no encouragement for physical activity (Photo: U2 S41).

Participants also took pictures of wide spaces and hallways within the universities, which completely lack encouragement for physical activity (G: Figure 7). Participants regard this as “lost” space and would appreciate inspiration for physical activity.

### Sedentary Behavior During Lectures and Learning Is Taken for Granted—Narrow Spaces Dispel Ambitions for More Movement

**Figure 8:**
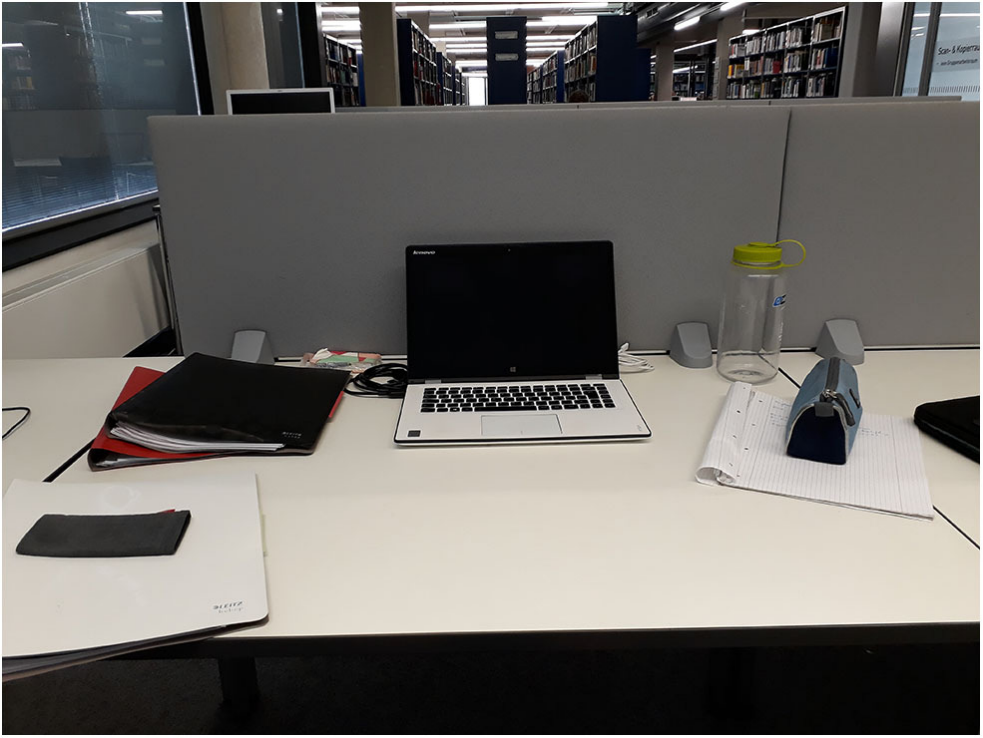
Students use a lot of equipment in library, e.g., laptops and books. There are no or very limited storage possibilities, which prevents them from getting up and moving during breaks (Photo: U2 S33).

Within libraries, sedentary behavior is considered inevitable by the participating students. The library rooms themselves offer no space for movement, according to the participants, and students avoid leaving the library (e.g., for active breaks) because they are afraid to leave their materials and computers behind (H: Figure 8).

You just don't know where to store your books. You are hardly encouraged to get up and go outside during a break, because you have to watch your stuff. (U1 FG1)

In both universities, stand-up tables are available within the library, but several barriers prevent their regular use: these tables are scarce, occupied by others most of the time, and/or often hidden in corners and thus not easily found. Moreover, stand-up tables are not adjustable to a sitting position. Students working on these tables are thus obligated to remain standing the entire time, which is exhausting for them. The participants suggest a more flexible solution.

Likewise, the participants explain that they feel obliged to stay seated during lectures. Most lectures last 1.5 h, and even if lecturers make a 5- or 10-min break in the middle of this period, the long narrow rows of chairs will render it difficult for students to leave the lecture hall for a short walk, according to the participants.

## Discussion

### Principal Findings

The photo voice study revealed that in neither of the two universities studied, the vast potential for increasing everyday physical activity on the University campus was exploited. According to the participants, the extensive green spaces between campus buildings offer no cues for movement: neither fixed nor mobile equipment for games or activities is (easily) available, and attractive walking trails are lacking or well-hidden. Instead, the lawns and meadows are used for sitting and relaxing. It became clear that cyclers struggle to find parking and storage options for bikes and biking gear as well as ramps and pathways free of steps when moving around campus, discouraging them from taking the bike to the campus in the first place. Restricting the options for physical activity (e.g., slackline parks) to the sports centers, as is the case in both universities, was considered both a physical and a psychological barrier to utilizing these offers. The participants did not feel well informed about many aspects regarding physical activity options on the campuses, e.g., they did not know the usage policy of lawns, or were not aware of options or events offered by the sports centers. The participants made many suggestions how the outdoor areas on campus could be changed for the better.

They were less optimistic when discussing the indoor situation. They reported how they felt obliged to keep sitting during lectures and learning time, and doubted that options for physical activity could be improved in the narrow spaces of lecture halls and libraries. The architectural design of staircases within buildings was regarded as mainly responsible for using—or not using—the elevators instead of walking. On the other hand, the participants pointed out that the wide hallways, foyers and courtyards, which were present all over the two campuses, could be put to better use with regard to physical activity. It became clear in the focus groups that the Photovoice project raised the participants' awareness of how the context influences their movement behavior, and helped them come up with solutions to make physical activity easier for students of their respective universities.

### Strengths and Weaknesses

Generally, the study design proved adequate for the focus of the study. The pictures helped the researchers get a more thorough insight into students' lives, and also helped the participants remember all the topics with regard to physical activity during the group discussions, as they served as a reminder. On the other hand, discussing photographed scenes and situations also prompted conversations about general topics and problems that were not linked to physical activity, e.g., complaining about construction sites on campus or malfunctioning doors; this required a clear focus and rigor of the facilitator.

We succeeded in recruiting students from a range of study programs, and could thereby capture heterogeneous views on physical activity on the campus. Nevertheless, it is not representative for the total number of students enrolled in those study courses. There was a slight predominance of students from sport related subjects (U1) and psychology (U2), respectively. Moreover, there were slightly more female participants. As participants were not only asked to take photos, but also to participate in a focus group discussion, outgoing and talkative people might have been attracted more; reserved and less talkative individuals may have been discouraged from participating.

A bias of the results may arise from the fact that the Photovoice project was conducted in summertime. This may have increased the participants' awareness of and focus on outdoor areas and outdoor activities, at the cost of the indoor context. For a more thorough understanding of the campus environment and its influence on physical activity, it may be advisable to perform additional data collections in winter. Still, students spend a lot of time indoors also in summer, e.g., during lectures, while learning in the library, or eating in the canteen; therefore, there was a substantial number of pictures taken inside the buildings that served as an adequate basis for discussions in the focus groups.

The study was conducted in two campus universities. The findings pertain to specific, unique built environments and cannot simply be transferred to other (campus) universities. It is interesting, however, that there were many similarities in both two locations, e.g., as to the usage of green spaces and hallways, the role of the sports center, sedentary behavior during lectures, and barriers for cyclers. Therefore, the results may serve as a catalog of potential contextual factors which may be worth considering when planning interventions for physical activity, as green spaces, sport centers, libraries and staircases can be found in any University.

### Comparison With Other Studies

There are only a few studies using a Photovoice approach to explore the setting of a University campus with regard to physical activity. Joy et al. used photo elicitation with volunteer University members (students *n* = 11, employees *n* = 14) to identify healthy eating and active lifestyle barriers and supports. Similar to our findings, participants reported that on campus, physical activity was principally possible, but dysfunctional (e.g., overgrown) walkways were regarded as barriers (18).

Deliens et al. focused on determinants of physical activity and sedentary behavior in Belgian University students using semi-structured focus groups. Unlike our findings, lack of time was named as the most important barrier to physical activity. Besides that, findings mainly corresponded with ours, e.g., lack of information on sports offers, or access to sports gear (27). This study has not used a Photovoice approach to contribute to the focus group discussion. Therefore, participants may have neglected factors connected to architectural design or layout of the University; in our study, students claimed that taking the pictures has raised the awareness of those environmental barriers/ facilitators.

In a study employing Photovoice sessions with female Hispanic adolescents, the participants identified some barriers to physical activity in the built environment of the community; these referred mainly to unaesthetic features (e.g., dirt, graffiti, vandalism), perceived lack of safety, or poor public transport to sports facilities (28). These aspects were not brought up by the participants of our study, hinting at the privileged status of the selected University campuses as compared to some (socially disadvantaged) neighborhoods.

### Implications for Policy and Practice

The Photovoice study helped identify starting points for environmental interventions that could foster physical activity among campus students. Creating and/or signposting attractive walks (including information on length of footpaths and time needed to walk along these paths) may “nudge” students to stroll over campus for talking with each other and/or winding down in breaks, rather than sitting. In addition, students may be more inclined to cycle to classes when campuses offer better options for moving around by bike, and for storing bikes and gear. Indeed, many studies have shown that creating new infrastructure for walking and cycling, including bike parking opportunities, were related to increased physical activity (29), although these data mainly refer to community and city designs. Few studies have focused on University campuses. Horacek et al. (30) analyzed the infrastructure of 13 US-American college campuses and showed that walkability and bikeability of the campus (i.e., the safety, quality and comfort of paths) was related to college students' physical activity. A study in a Hongkong University demonstrated that increasing and repairing the pedestrian networks improved the students‘ walking behavior (31).

In both studied universities, the outdoor areas dispose of vast green spaces that were currently mainly used for sitting and relaxing; cues for movement were reported to be missing. The Photovoice participants emphasized that easily accessible activity areas, as well as a sports equipment sharing program, could encourage students to get physically active in breaks. According to a number of studies in schools, improving playground structures and designs as well as providing game equipment could increase the moderate or moderate-vigorous physical activity in children during recess, although the results were mixed (32). It is not clear if these results can be transferred to the University setting and University students, respectively; experimental studies on the effect of gaming equipment and exercise facilities in universities are lacking. It is interesting that the activity options that the participants suggested included gaming and “fun” sports (i.e., slacklines, table soccer, or bouldering sites) rather than “classical” fitness or team sports (e.g., outdoor workout equipment, soccer, basketball). This aspect would be worth exploring further before deciding on offers and changes in infrastructures, for example by performing a survey among University students on their preferred activity and exercise types. A survey among Emirati University students revealed that indeed the vast majority preferred activities “with a fun element,” although the activities in question were not restricted to the campus setting (33). Probably, nonathletic and athletic students may also differ with regard to their activity preferences.

Whereas, breaks and recreation between classes and learning phases provide the main opportunities for University-based physical activity, interventions may also target the sedentary behavior prevalent in lecture halls and libraries. The participants stated they felt uneasy leaving their textbooks, notes and laptops behind. Therefore, on-site exercise measures seem appropriate, for example sit-stand desks, or activity breaks in classrooms. In a review on standings desks in school classrooms, Minges et al. (34) show that those desks reduce sitting time, whereas the results with regard to physical activity are mixed. Reducing sedentary time may be even more significant in the University sector, though, where students sit even longer than in school. Activity breaks can reduce a prolonged sitting time and increase physical activity, as several studies among school children have shown (35–38). To our knowledge there are no studies focusing on University students. Therefore, we do not know if activity breaks can be implemented in classes and lectures of universities; reluctance of lecturers to offer exercises in breaks, and/or reluctance of students to participate may be barriers.

The Photovoice study could highlight a range of environmental factors—indoors and outdoors—which influence sedentary behavior and physical activity of University students. This catalog may serve as a base for aspects to be considered when analyzing the campus setting before implementing measures. Systematic approaches to gather information pertaining to the campus environment are scarce. Horacek et al. (39) developed a tool to assess the built environment of college campuses; its components include bike racks, stairwell direction prompts, exercise spaces, available equipment, and quality and safety of paths. According to the results of our study, such an audit should be supplemented with factors relating to indoor situations in buildings and libraries, storage facilities, as well as more general aspects pertaining to the layout of campus grounds (stairs, rondels etc.) (39). Murphy et al. (40) published a study protocol indicating that there is work underway to create a comprehensive audit tool for examining the environment, provision, and support offered by Irish universities for students' participation in physical activity. This may give further hints as to environmental factors relating to physical activity of students.

## Conclusion

The Photovoice method proved to be an adequate approach for thoroughly analyzing the environmental factors that influence physical activity and sedentary behavior, seen through the lens of those individuals spending a lot of time in this environment. It helped identify a wide range of starting points for health-related interventions. Future research needs to focus on questions around how students can be actively involved in planning and advocating for (structural) interventions that change campuses to be healthier places which support movement and active lifestyles. Analyzing the effectiveness of such interventions will also warrant further research.

## Data Availability Statement

The raw data supporting the conclusions of this article will be made available by the authors, without undue reservation.

## Ethics Statement

The studies involving human participants were reviewed and approved by Ethics Committee of the University of Regensburg (18-896-101). The patients/participants provided their written informed consent to participate in this study.

## Author Contributions

JvS conducted the focus groups, coded and analyzed the material, and was a major contributor in writing the manuscript. JL set up the study design, commented on the interview guide, and was a major contributor in writing this manuscript. JR set up the study design, developed the interview guide, and conducted focus groups. JC conducted focus groups in one of the universities. JH and ST helped to set up and conduct the focus groups in one of the universities. All authors contributed to the article and approved the submitted version.

## Conflict of Interest

The authors declare that the research was conducted in the absence of any commercial or financial relationships that could be construed as a potential conflict of interest.

## Abbreviations

N: Number
FG: Focus Group
U1: University 1
U2: University 2
S: Student.
